# Measuring cataract outcomes

**Published:** 2022-12-16

**Authors:** John Buchan

**Affiliations:** 1 Programme Director: MSc Public Health for Eye Care, ICEH, London School of Hygiene & Tropical Medicine and Clinical Lead: RCOphth National Ophthalmology Database Cataract Audit, Leeds, UK.

**Measuring the outcomes of cataract surgery can drive improvement and patient satisfaction. But is visual acuity measurement the only way?**

**Figure:**
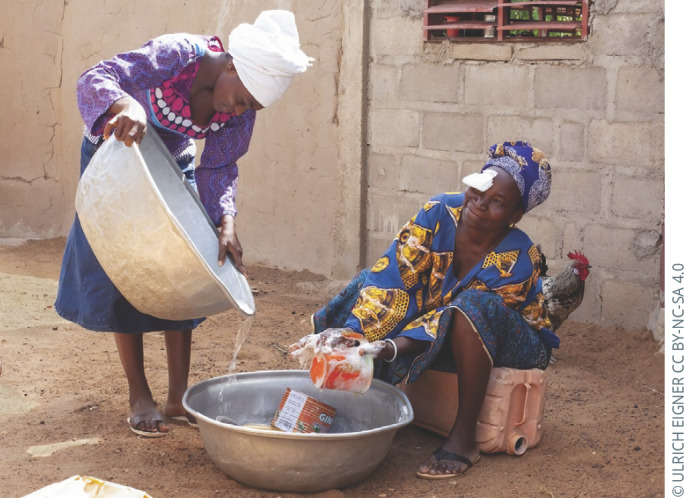
A cataract patient carrying out her daily tasks with ease after a successful operation. **BURKINA FASO**

Cataract surgery can be a frightening prospect for many patients. Hearing from others in their community who are happy with the results can have a significant impact and increase the overall uptake of cataract surgery in that community. To achieve this, we need to deliver cataract services that are successful in the opinion of the most important people: the patients.

But how can we know whether our patients are happy, and what matters to them?

A patient-centred approach has two components:

1. Patients’ **experience** of the cataract service before, during and after surgery. This can include comfort/pain, cleanliness, communication, and many other aspects of care. We cover this in more detail in another article in this issue.
2. The **visual outcome** of surgery, which surgeons need to know so they can check their surgery is of good quality. This will be the main focus of this article.

## Visual outcome

The success or failure of cataract surgery has traditionally been assessed by measuring a patient’s presenting visual acuity after surgery.

Visual acuity is an essential benchmark for the quality of cataract surgery. We should all aspire to meet the WHO’s new recommendation that 80% of eyes operated on should have a presenting visual acuity of 6/12 or better after surgery.^1^ In fact, by measuring and publicly reporting the visual acuity outcomes of cataract surgery, the United Kingdom’s National Health Service was able to significantly improve outcomes by introducing a National Ophthalmology Database Cataract Audit in 2014. Likewise, tools such as the free BOOST cataract app (**https://boostcataract.org**) allow surgeons in low- or middle-income settings to monitor cataract outcomes and receive feedback without incurring additional costs.^2^ Publishing these data publicly can improve outcomes and boost public confidence – which in turn improves the uptake of cataract surgery.^3^

In most low-income settings, patients tend to come for surgery when their cataract is already advanced. For them, a presenting visual acuity outcome of better or equal to 6/12 (the new World Health Organization benchmark) is highly satisfactory.

However, in high-income settings, the excellent visual acuity outcomes of cataract surgery, combined with the availability and affordability of surgery, has led to early uptake of services. For example, at least a third of patients undergoing cataract surgery in the UK have pre-operative visual acuity of 6/12. For them, visual acuity is a less useful indicator of the success of surgery.^3^

Visual acuity is usually measured by asking patients to read black letters on a white background at six metres – a task that few patients ever need to do in real life. Patients with higher pre-operative visual acuity will be more interested in their visual function: how the operation has improved their ability to do everyday tasks such as cooking, reading, or driving. Distance visual acuity alone, therefore, is not a perfect measure of success for, as it doesn’t tell us much about the patient’s perspective – how they perceive their own vision and visual function, and the impact on their quality of life.

Patient-reported outcome measures (PROMs) are a potential solution to this dilemma.^4^ PROMs are short questionnaires given to patients before and after surgery to ask about their own perception of their vision and the impact of their vision on their quality of life; this is expressed as a numerical score.

Although perception of vision and quality of life are subjective (i.e., individual to each patient), PROM questions are developed through a robust process of research, testing, and mathematical analysis, which means that the scores produced when the questionnaire is administered before and after surgery can provide a reliable measurement of the improvement experienced by each patient. Creating PROMs requires the input of patients during development to ensure they consider patients’ visual needs, which will vary depending on factors such as patients’ level of literacy or the need to be able to drive.

PROMs put patients’ perception of their own vision at the centre. This encourages clinicians to listen to patients and helps them to understand how patients’ vision impacts their quality of life, which in turn permits health care professionals to develop services that meet the needs and expectations of patients – a very desirable outcome.

**The importance of monitoring quality**

Faced with a high prevalence of cataract blindness, increasing the quantity of surgery is often essential. However, this needs to be accompanied by monitoring the quality of what is being done.

*For further reading on outcome monitoring see* **https://bit.ly/CEHJ-cat**

## References

[1] Keel S Muller A Block S, et al. Keeping an eye on eye care: monitoring progress towards effective coverage. Lancet Glob Health 2021; 9 (10): e1460–e4. 34237266 10.1016/S2214-109X(21)00212-6 PMC8440222

[2] Congdon N Suburaman G-B Ravilla T, et al. Transforming research results into useful tools for global health: BOOST. Lancet Glob Health. 2016, 4:e96. 26823227 10.1016/S2214-109X(15)00267-3

[3] Yoshizaki M Ramke J Zhang JH Aghaji A Furtado JM Burn H Gichuhi S Dean WH Congdon N Burton MJ Buchan J. How can we improve the quality of cataract services for all? A global scoping review. Clin Exp Ophthalmol. 2021;49(7): 672–685. 34291550 10.1111/ceo.13976 PMC7618289

[4] Braithwaite T Calvert M Gray A Pesudovs K Denniston AK. The use of patient-reported outcome research in modern ophthalmology: impact on clinical trials and routine clinical practice. Patient Relat Outcome Meas. 2019, 10:9–24. **https://bit.ly/CEHJ-prom** 30774489 10.2147/PROM.S162802 PMC6352858

